# Con-DAM: Simultaneous measurement of food intake and sleep in *Drosophila* at the single fly resolution

**DOI:** 10.17912/micropub.biology.001200

**Published:** 2024-06-30

**Authors:** Breanna Beard, Abigail Bohn, Mubaraq Opoola, Dae-Sung Hwangbo

**Affiliations:** 1 Department of Biology, University of Louisville, Louisville, Kentucky, United States

## Abstract

Sleep and feeding are conserved behaviors across many taxa of the animal kingdom and are essential for an organism's survival and fitness. Although *Drosophila* has been used to study these behaviors for decades, concurrent measurement of these two behaviors in the same flies on solid media has been a challenge. Here, we report Con-DAM, which enables simultaneous quantification of food intake and sleep/activity at the single fly resolution. Since Con-DAM integrates the Con-Ex (Consumption-Excretion) assay and the DAM (Drosophila Activity Monitor), two widely used tools to quantify food consumption and sleep/activity in flies into a single unit, we expect Con-DAM to serve as an easy method for various purposes that require quantifying food consumption and sleep/activity in the same individual flies.

**Figure 1. Simultaneous measurement of food consumption and sleep using Con-DAM:**
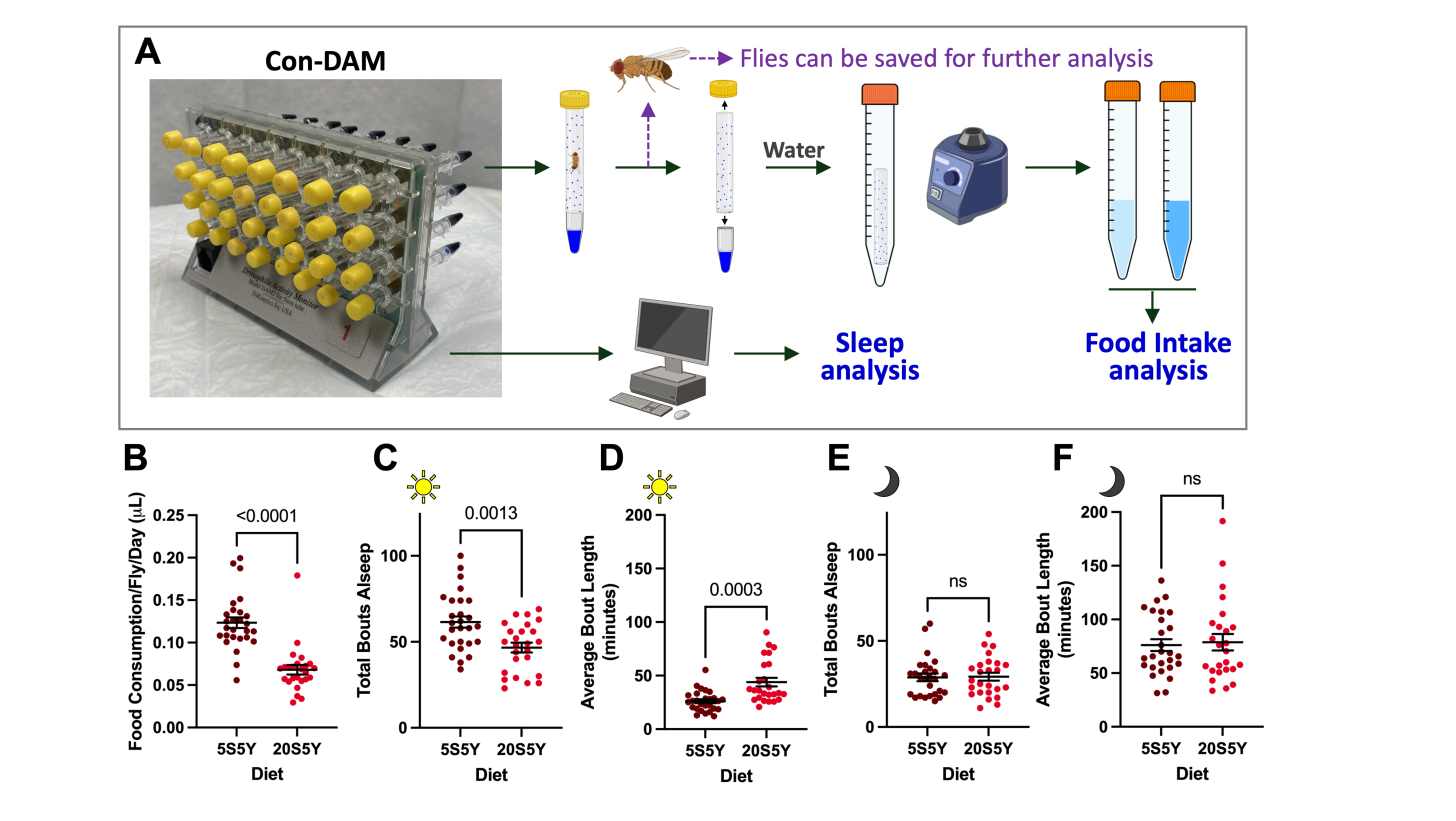
**(A)** Illustration and workflow of the Con-DAM method: Single flies are housed in individual glass tubes containing experimental food mixed with 1% blue dye (FD&C Blue #1) in a PCR tube. Sleep and food consumption are measured separately without sacrificing the flies. Refer to the methods section for details. For figure 1B-F, flies were placed on high sugar (20% sugar, 5% yeast, 20S5Y) or low sugar (5% sugar, 5% yeast, 5S5Y) and were loaded into the DAM system. Thereafter, sleep and feeding behaviors were quantified. Excreta was collected by placing the glass tube in a falcon tube and vortexing with water. **(B)** Total food consumption per fly per 24 hours over the duration of the experiment on low sugar (n=27) and high sugar (n=25). **(C-D)** Sleep behavior of flies on high and low sugar over a 72-hour period during the daytime (12 hours of light on; a total of 36 hours during the experiment) conditions. **(E-F)** Sleep behavior of flies on high and low sugar over a 72-hour period during the nighttime (12 hours of light off; a total of 36 hours during the experiment) conditions. Total Bouts Asleep: The number of sleep bouts (as a period of five or more minutes of inactivity measured by infrared beam breaks) during the phase of the experiment. ABL: Average Bout Length of sleep for the phase of the experiment. Error bars indicate standard error of the mean. Statistical significance was determined using an unpaired t-test or Welch's t-test.

## Description

Feeding and sleep are essential behaviors that are necessary to sustain life in nearly every organism of the animal kingdom, including the fruit fly, *Drosophila melanogaster.* These behaviors regulate a diverse set of biological processes, such as development, metabolism, fecundity, and lifespan (Kayser & Biron, 2016; Potdar et al., 2018; Thompson et al., 2020; Xu et al., 2011; Xu et al., 2008; Yurgel et al., 2018) . They are also strongly influenced by the circadian clock, energy demands, and dietary composition (Catterson et al., 2010; Chatterjee & Hardin, 2010; Pool & Scott, 2014) .

Since sleep and feeding behaviors are homeostatic but mutually exclusive where one must be suppressed when the other is prioritized to restore the organism to its optimal level, tight regulations must operate between them (McDonald & Keene, 2010) . Many evolutionarily conserved neuronal and hormonal circuits that regulate both feeding and sleep have been discovered in flies, including Neuropeptide F (homologous to mammalian Neuropeptide Y) and dopamine signaling (Andretic et al., 2005; Chung et al., 2017; Marella et al., 2012) , making the fly an attractive model organism to understand the nature and mechanisms driving these behaviors.

Similar to sleep in mammals, sleep in flies is characterized by a postural shift, decreased responsiveness to external stimuli, rebound sleep following sleep deprivation, and suppression of sleep during starvation (Borbely, 1982; Cirelli & Bushey, 2008; Hendricks et al., 2000; Keene et al., 2010). Measurement of sleep in flies can be achieved through various methods, including video tracking, measurement of local field potentials, and whole body calorimetry (Shafer & Keene, 2021) . However, one of the most widely used and accessible methods for measuring sleep in fruit flies is the *Drosophila* Activity Monitoring (DAM) system. The DAM system records the gross movement patterns of flies when they break the path of an infrared beam in small tubes in which they are housed. Sleep is defined as a period of quiescence (lack of infrared beam breaks) for five or more minutes (Hendricks et al., 2000; Shaw et al., 2000; Zimmerman et al., 2008) . The DAM system is a powerful high throughput tool useful for assaying sleep, activity, and starvation resistance of single flies, making this method well-suited for large scale genetic or chemical modifier screens.

Feeding in *Drosophila* is achieved and modulated through the coordination of specialized neurons, neuromodulators and peptides, nutrient sensors, and synchronization with environmental cues (Itskov & Ribeiro, 2013; Pool & Scott, 2014) . Similarly to sleep, feeding in flies is strongly influenced by social experience, dietary composition, and metabolic state (Carvalho et al., 2005; Garlapow et al., 2015; Li et al., 2021; May et al., 2019; Shell & Grotewiel, 2022) . Flies consume food on the micro-scale, making the accurate measurement of feeding behavior technically and conceptually challenging. To overcome this challenge, many methods have been developed to quantify feeding indirectly (Reviewed in (Deshpande et al., 2014) ). Among them, the consumption-excretion (Con-Ex) and the excreta quantification (Ex-Q), which involve measuring excreta after feeding flies with dye-labeled food, offer an easy and non-invasive method for measuring food consumption (Shell & Grotewiel, 2022; Shell et al., 2018; Wu et al., 2020) .

Despite the reciprocal relationship between sleep and feeding behaviors, these behaviors have traditionally been studied separately, presumably due to technical challenges in quantifying them concurrently in the same flies. Here, we report a newly developed method termed Con-DAM, which integrates the Con-Ex and the DAM systems into a single platform for simultaneous measurement of sleep and food consumption at the single fly resolution. Briefly, the DAM component of the Con-DAM records sleep and activity behaviors of individual flies housed in glass tubes, while the Con-Ex component of the Con-DAM measures food consumption through the use of solid food media supplemented with 1% blue dye (FD&C Blue #1). Since the dyed food is aliquoted in PCR tubes and plugged outside of the glass tubes for the DAM component of the Con-DAM, flies' excreta are collected only inside of the glass tubes and measured to quantify food consumption ( Figure 1A, please see methods for details ).

To validate Con-DAM, we employed high sugar (20% sucrose 5% yeast, 20S5Y) and low sugar (5% sucrose 5% yeast, 5S5Y) diets, as dietary sucrose is known to alter both sleep and food consumption in flies (Brown et al., 2020; Carvalho et al., 2005; Linford et al., 2012; May et al., 2019) . In general, at higher dietary sucrose concentrations, food consumption and sleep bout numbers decrease, while sleep bout duration (or the number of long sleep bouts) tends to increase (Linford et al., 2012; May et al., 2019) . To test sleep and feeding behaviors using Con-DAM, young (3-5 days old) male flies were individually transferred into glass tubes housed in the DAM board using light CO _2_ anesthesia and were allowed to feed on the high or low sugar diet containing 1% blue dye for 4 days. As expected, flies fed the high sugar diet consumed significantly less food per day than flies fed the low sugar diet (Carvalho et al., 2005) ( Figure 1B, p < 0.0001). Similarly, the same flies fed on the high sugar had significantly less sleep bouts but a longer average bout length than the flies consuming the low sugar diet ( Figure 1C -F) especially during the day. These results validate that Con-DAM effectively quantifies food consumption and sleep under the conditions and time period used for the sleep measurement.

In this manuscript, we present Con-DAM, a simple integration of two independently developed tools into a single platform for measuring food consumption and sleep in the same individual flies on solid food without a time delay. Despite the limitation of DAM-based sleep measurement where sleep is extrapolated from the gross activity of flies (Zimmerman et al., 2008) , we anticipate that Con-DAM will be an easy straightforward option for researchers investigating the relationship between sleep and food consumption, including large scale genetic and chemical modifier screens for sleep and feeding behavior. However, Con-DAM may not be ideal for long term measurement of feeding and sleep, as accumulated excreta could cause flies to get trapped or stuck on the sides of vials unless flies are transferred to new glass tubes. Another limitation of Con-DAM is that unlike its sleep counterpart, food consumption cannot be continuously measured, making it challenging to understand the dynamic temporal relationship between feeding and sleep behaviors. These limitations can be partially overcome with the Activity Recording Capillary feeder (ARC), where both sleep and feeding behaviors are continuously monitored using high-resolution visual tracking (Murphy et al., 2017) . However, since the ARC only allows for liquid diets, we recommend that researchers utilize a combination of Con-DAM, ARC, and Con-FLIC, which we recently reported to concurrently measure various feeding behaviors and food consumption on liquid food (Opoola et al., 2024) , depending on experimental conditions and purposes. Although we used DAM2 as the base platform, other activity monitors such as the DAM5H-4, a multi-beam activity monitor, can also be easily adopted to the Con-DAM platform.

Overall, despite some challenges, we expect that Con-DAM can serve as a simple tool that can be easily adopted by investigators to understand the relationship between feeding and sleep without sacrificing flies which can be saved for subsequent biochemical and other behavioral assays.

## Methods

***Fly Strains, Husbandry, and Diets***

Male *w1118* flies (Bloomington Stock #5905) were used for all experiments. Flies were reared on Bloomington Standard Cornmeal medium (Genesee Scientific, Cat#: 66-113) supplemented with 1% (w/v) Brewer's Yeast (MP Biomedicals) under 12:12 LD cycles at 25°C and 40%~60% relative humidity. For the Con-DAM assay, 20S5Y (20% sucrose and 5% yeast in w/v) and 5S5Y (5% sucrose and 5% yeast in w/v) diets mixed with 1% blue dye (FD&C Blue #1) were used.

***Con-DAM Configuration***

The Con-DAM combines and modifies the DAM system commercially available from TriKinetics (TriKinetics, Waltham, MA) and the Con-Ex method described in Shell et. al. (2018) (Shell et al., 2018) . DAM2 boards, glass tubes, auxiliary supplies for DAM system, and software (DAMSystem311 and DAMFileScan113) were acquired from TriKinetics (www.trikinetics.com). The DAM component of the Con-DAM consists of a DAM2 board, which houses 32 individual flies in small glass tubes (5mm diameter by 65 mm length). An infrared beam crosses the midpoint of each tube and is interrupted when the fly crosses the midpoint of the tube, allowing for the quantification of sleep and activity. DAMSystem311 software acquires the activity movements from the DAM2 boards, while DAMFileScan113 converts raw monitor files to a format compatible for analysis. The Con-Ex component of the Con-DAM consists of a modified version of the Con-Ex assay described in Shell et. al., 2018 (Shell et al., 2018) . To achieve this, we used solid media (sucrose/yeast diet) mixed with 1% (w/v) FD&C Blue #1 dye. Using a pipette, approximately 100 mL of dyed sucrose/yeast media was aliquoted into a PCR tube and placed directly onto one end of the glass tubes. The other end of the glass tube was closed with a yellow cap with small holes for ventilation. In principle, the dyed food in PCR tubes does not touch the glass tubes, and only the flies' excreta inside the tubes are collected for food measurement. To collect the excreta on the sides of the glass tubes, the PCR tube was removed and any excess blue dye on the outside of the tube, which may occur during the preparation steps, was removed with clean wipers such as Kimwipes®. At this step, flies can be saved or frozen for further analysis. The tubes were placed in a 15 mL falcon tube filled with 1.5 mL of deionized water. The falcon tube was inverted and vortexed to cleanse the dye from the glass tube out into the falcon tune ( Figure 1A ). The amount of water used to cleanse the dye can be adjusted depending on the size of the falcon tubes and other experimental conditions.

***Con-DAM Assay***

Following ~ 48 hours of post-eclosion mating, young (3-5 days old) male flies (~72-hour cohorts) were separated into individual glass tubes containing 5S5Y or 20S5Y diet and placed into the Con-DAM board using light CO _2_ . Flies were measured in Con-DAM board for ~4 days for sleep/activity and feeding. They were given ~12 hours to recover from CO _2_ before sleep analysis. Sleep and activity were analyzed for three days, while feeding was recorded for a total of four days.

***Statistical analysis and visualization***

The feeding data collected from Con-DAM were analyzed utilizing the protocol described in (Shell et al., 2018) with minor modifications. The concentration was determined via spectrophotometry at an absorbance of 630 nm. Optical density was determined by extrapolating on a standard curve. The raw data collected from the DAM component of the Con-DAM were processed with Counting Macro (Version 5.19.9) using the protocol described in (Pfeiffenberger et al., 2010) . Counting Macro is an Excel-based computer program developed to process data generated by the DAM system. Flies with less than 6 beam breakings during the last experimental day and stuck to the glass tubes presumably due to condensation were removed from analysis. Statistical analysis (unpaired t-test or Welch's t-test) and figures were produced using GraphPad Prism (Version 10) or Microsoft Excel (Version 16.81).
